# THE DOCTOR-PATIENT RELATIONSHIP, PERSONALITY, MOOD, AND FUNCTIONING IN OLDER ADULTS

**DOI:** 10.1093/geroni/igz038.578

**Published:** 2019-11-08

**Authors:** Lauren S Atlas, Richard Zweig

**Affiliations:** 1 Yeshiva University, Bronx, New York, United States; 2 Yeshiva University, New York, United States

## Abstract

Personality pathology has been tied to mental and physical health in older adulthood. Less is known regarding the combined impact of personality and the doctor-patient relationship on mental health outcomes. This study examined relationships between personality, mood, and trust in physicians. Participants (N=170) were a sample of primary care older adults ages 60-99 (M = 70.73, SD = 7.054) who completed self-report measures of personality traits (NEO-FFI), processes (IIP-PD-25), depression (GDS-30; PHQ-9), social adjustment (SAS-SR) and trust in one’s physician (GTIP). Medical burden data (CIRS) were retrieved from medical records. After adjusting for relevant covariates such as age, perceived health, cumulative illness burden, and income security there were several significant predictive relationships. In combined models more neuroticism (NEO-N, ß = .082, p < .000) and lower trust (GTIP, ß = -.025, p = .014) but not agreeableness (NEO-A, ß = -.006) or interpersonal problems (IIP-25, ß = .254) predicted depression. In combined models, higher neuroticism (NEO-N, ß = .018, p < .000) and interpersonal problems (IIP-25, ß = .186, p = .002) but not agreeableness (NEO-A, ß = -.003) or trust (GTIP, ß = -.002) predicted social adjustment. The results are consistent with previous findings that neuroticism predicts both depression and social adjustment in older adults. In addition, lower trust augmented neuroticism to predict depression. Results suggest that apart from general personality risk factors, situational personality processes such as trust in physicians may affect mood state, whereas personality processes such as interpersonal problems contribute to longer term functional impairment.

